# Winter food sources for the Grey-headed Woodpecker (*Picus canus*) in South Korea using faecal analysis in nest boxes

**DOI:** 10.3897/BDJ.14.e173032

**Published:** 2026-01-14

**Authors:** Sang-Yeon Lee, Eui-Jeong Hong, Eun-Jeong Lee, Junseok Lee, Hacheol Sung

**Affiliations:** 1 National Ecosystem Survey Team, National Institute of Ecology, Seocheon, Republic of Korea National Ecosystem Survey Team, National Institute of Ecology Seocheon Republic of Korea; 2 Incorporated association ECO Korea, Goyang, Republic of Korea Incorporated association ECO Korea Goyang Republic of Korea; 3 Department of Biological Sciences, Institute of Sustainable Ecological Environment, Chonnam National University, Gwangju, Republic of Korea Department of Biological Sciences, Institute of Sustainable Ecological Environment, Chonnam National University Gwangju Republic of Korea

**Keywords:** Anacardiaceae, faecal analysis, Formicidae, nest box, *Picus canus*, relative frequency, specialist, winter

## Abstract

The Grey-headed woodpecker (*Picus canus*) inhabiting South Korea is an insectivorous bird species that favours ants. However, during winter, most insects enter a dormant stage, reducing their availability as food sources. This may prompt *P. canus* to change its primary food sources. To verify this hypothesis, we fabricated and installed 91 nest boxes in forests in Seocheon-gun, Chungnam Province, considering the body size of *P. canus*. From these, we collected faeces samples totalling 94 conical tubes of 1.5 ml each, obtained from 17 nest boxes used by *P. canus* as roosting sites. The collected faeces samples were examined under a microscope and their relative frequencies were calculated at the family or order level. Formicidae exceeded 70%, confirming it as the primary food source for *P. canus* during the breeding season and also in winter. Anacardiaceae fruits accounted for approximately 16%, significantly lower than Formicidae, but still considered a major winter food source for *P. canus*.

## Introduction

Birds typically employ a feeding strategy to select optimal food sources to minimise foraging time and maximise energy intake, enhancing their survival chances (Stephens and Krebs 1986, Krebs and Davies 1993). Understanding these feeding strategies is crucial for comprehending avian ecology (Litvaitis 2000) and serves as fundamental information for species conservation and habitat management (Moreby and Stoate 2000, Fukui and Agetsuma 2010). As food sources of birds vary according to the season (Foster 1977, Török 1990), it is necessary to identify these food sources into breeding and non-breeding seasons or by seasonal distinctions (Pechacek and Kristin 2004).

Methods for analysing bird food sources include: i) direct observation (e.g. Kleintjes and Dahlsten (1992), Read (1994), Yang et al. (2009), Lee et al. (2018)); ii) neck-collars (e.g. Koo and Won (1986), Poulsen and Aebischer (1995), Grim and Honza (1996)); iii) stomach contents analysis (e.g. Otvos and Stark (1985), Mellott and Woods (1993), Hess and James (1998), Pechacek and Kristin (2004), Kim et al. (2018)) and iv) faecal analysis (e.g. Kojima and Matsuoka (1985), Galbraith et al. (1993), Stoate et al. (1998), Joo et al. (2016)). Amongst these, faecal analysis identifies food sources without causing harm to the individual, based on undigested fragments that are excreted, thus minimising the potential for ethical concerns (Exnerova et al. 2003, Moorman et al. 2007). While there is an argument that easily digestible food sources may be underestimated compared to that ingested (Robinson and Stebbings 1993, Litvaitis 2000), some studies suggest no significant differences (Waugh 1978, Matsuoka and Kojima 1985). The neck-collar method or stomach content analysis allows for the verification of short-term food sources only. In contrast, faecal analysis examines a more comprehensive range of food sources collected over longer periods and in broader geographical areas (Moreby and Stoate 2000).

The Grey-headed Woodpecker (*Picus canus*) is a widely distributed species occurring from Europe through Siberia to Northeast Asia. According to the IOC World Bird List (v15.1; Gill et al. (2025)), the species comprises 10 recognised subspecies. The population occurring in South Korea belongs to *P. c. jessoensis*, which is distributed across the Korean Peninsula and parts of Japan. This species is known to primarily feed on ants during the breeding season (Angelstam and Mikusiński 1994). A study applying the neck-collars to the offspring of *P. canus* in South Korea identified food sources including beetles, ants, spiders, fruits of *Prunus* spp. and seeds of *Pinus* spp. Out of 7078 food items identified, 6896 (97.4%) were overwhelmingly dominated by ants (Koo and Won 1986). During the winter season, when numerous insect species, including ants, enter a dormant stage (Karr 1976), the availability of food sources decreases, potentially leading to shifts in the winter diet of *P. canus*. However, research on winter food sources related to *P. canus* in South Korea is limited.

This study aims to evaluate the winter food sources of *P. canus* through faecal analysis and to determine whether ants, the predominant food source during the breeding season, continue to be the primary food source in winter or whether there is a shift to alternative food sources. This information offers valuable insights into the species' ecology.

## Material and methods

**Faeces collection utilising nest boxes**

In wildlife studies, faeces have been collected using various approaches. Amongst them, two commonly cited methods are: i) collection from captured individuals (Moreby and Stoate 2000, Chung et al. 2015) and ii) collection from feeding or resting sites (Kojima and Matsuoka 1985, Choi et al. 2017). However, capturing adult *P. canus* individuals or observing their defecation behaviour in the field is challenging. Consequently, the above-mentioned methods appear unsuitable for collecting *P. canus*' faeces (Lee pers. comm.). Conversely, Matsuoka and Kojima (1985) collected winter faeces of *P. canus* from nest boxes in Japan and presented the resulting food source data. This implies that *P. canus* utilises nest boxes as roosting sites during winter and defecates within them. Therefore, we installed nest boxes tailored to the body size of *P. canus* to facilitate efficient faeces collection.

After reviewing various designs for woodpecker nest boxes through a nest box guidebook (du Feu 2003) and an online search (using the search term “woodpecker nest box plan”), a final design was selected. The chosen design features an entrance diameter of 55 mm, an entrance-to-floor height of 340 mm and an internal space measuring 160 mm × 160 mm. These nest boxes were constructed from 15 mm thick red pine wood, with wood chips on the floor. Thus, 91 nest boxes were completed.

**Figure 1.**
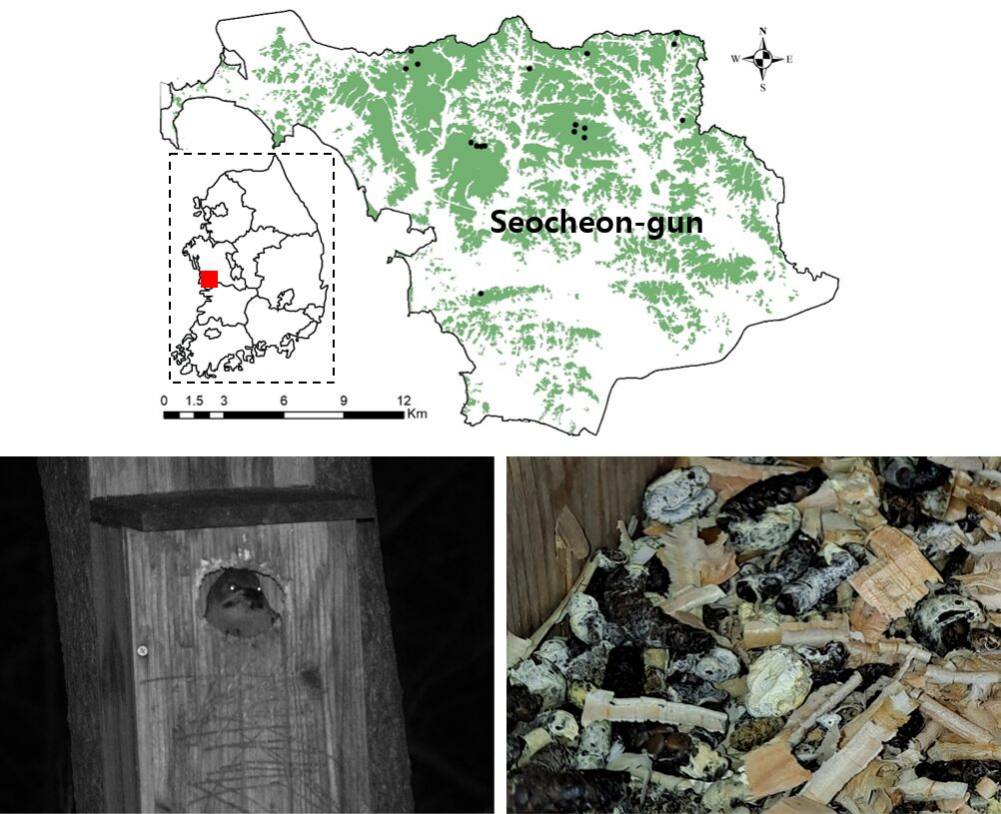
Black dots represent the positions of the nest boxes utilised as roosting sites by *Picus canus* (n = 17). Roosting behaviour of *P. canus* (bottom left) and faeces of *P. canus* inside the nest box (bottom right).

The nest boxes were installed at a minimum height of 3 m above the ground in the forests of Seocheon at the end of November 2020. Considering the ecological characteristics of *P. canus* living solitarily (Gorman 2014), the spacing between nest boxes was maintained at a minimum distance of 100 m. We monitored faeces in nest boxes weekly from December to February of the following year. When faeces were detected, we installed Force-12 unmanned sensor cameras (Spypoint, Canada) in front of the nest boxes or conducted observations from a distance one hour before sunset using an AT/ATS80HD 25×–50× scope (Swarovski Optik, Austria) to confirm the entry and exit of *P. canus*. Faeces found in nest boxes with confirmed use were collected (Fig. 1) and approximately half of each sample was placed into 1.5 ml conical tubes (n = 94) and preserved in 70% ethanol prior to freezing (Matsuoka and Kojima 1985, Pechacek and Kristin 2004, Chung et al. 2015).

**Food source identification and relative frequency calculation**

The liquid in the conical tubes was decanted and the faeces were manually broken apart and spread widely on to Petri dishes (Orłowski and Karg 2013). A stereomicroscope Leica M205C (Leica, Germany) facilitated the separation of insects and plants on a large scale. Identifying food sources from faeces involves estimation due to numerous fragments, posing a high risk of insect and plant identification errors without sufficient experience and skill (Holechek 1982, Moreby and Stoate 2000). Therefore, the separated fragments were photographed in 3D using a Leica DFC450 camera (Leica, Germany) attached to the microscope. They were then subjected to primary identification by taxonomic experts in each relevant taxon. In cases where images were deemed ambiguous during primary identification, three additional taxonomic experts were enlisted for secondary verification and an identification was accepted only when at least four experts reached the same conclusion. Food sources were classified up to the family level in the higher taxonomic rank and the order level if classification beyond that was not feasible.

We computed the relative frequency of each identified food source from individual conical tubes using the formula: relative frequency of a food source in one conical tube (%) = (1 / number of food source types identified in one conical tube) × 100% (Pechacek and Kristin 2004). As fragment-based faecal data do not allow reliable abundance estimation (Kervyn and Lobois 2008), only relative frequency comparisons were performed.

## Results

**Figure 2.**
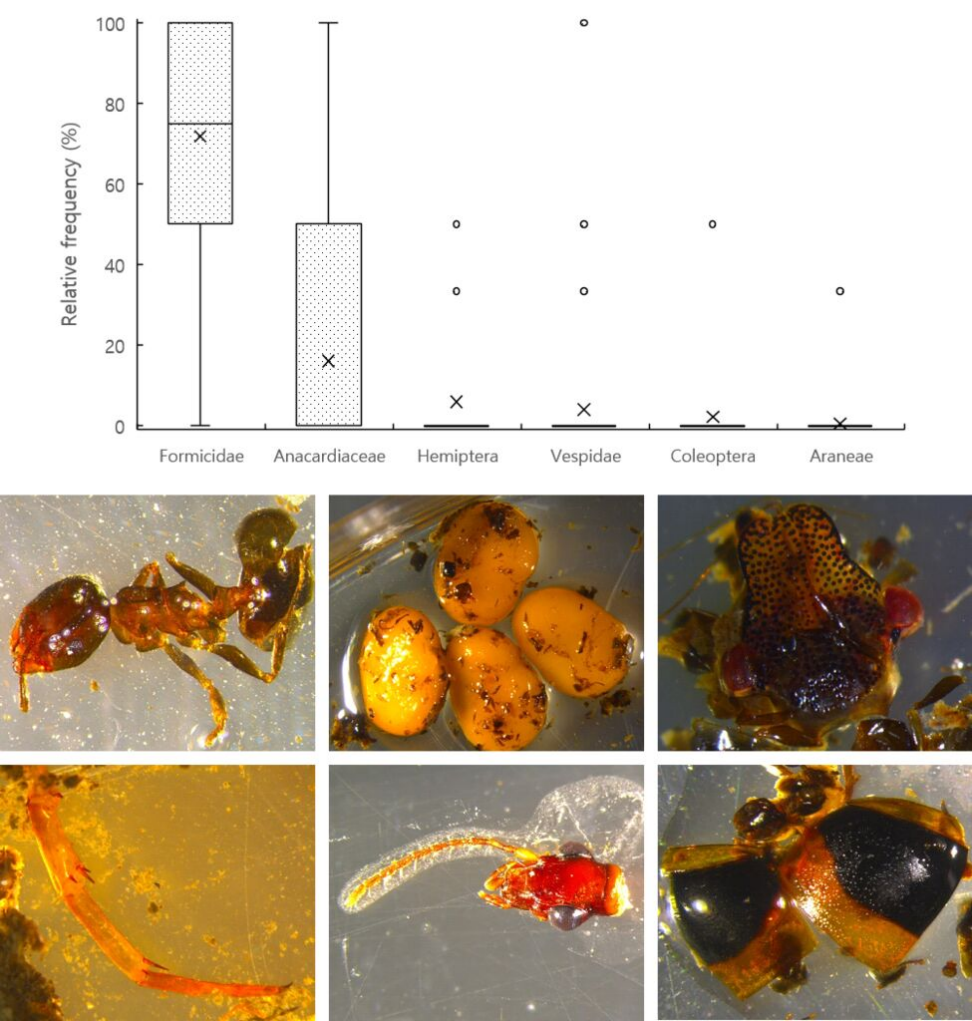
The relative frequencies of winter food sources are categorised by the higher taxonomic ranks of *Picus canus*. Box plots display the median, interquartile range (25% and 75% quartiles) and whiskers indicating minimum and maximum values, with × representing the mean values (n = 94). The images show the winter food sources of *P. canus* confirmed under a microscope. Starting from the middle left and moving clockwise: Formicidae, Anacardiaceae, Hemiptera, Vespidae, Coleoptera and Araneae.

Of the 91 nest boxes installed, 17 were confirmed to be used by *P. canus*, from which 94 faecal samples were collected. Formicidae showed the highest relative frequency (71.81 ± 3.08%), followed by fruits of Anacardiaceae (15.96 ± 2.57%). Other food categories, including Hemiptera (5.85 ± 1.49%), Vespidae (3.90 ± 1.54%), Coleoptera (2.13 ± 1.05%) and Araneae (0.35 ± 0.35%), occurred at much lower relative frequencies (all < 6%) (Fig. 2).

Due to the limited occurrence of the remaining taxa, statistical comparisons were restricted to Formicidae and Anacardiaceae, which together comprised the majority of food items. A Mann–Whitney U test, conducted using Python (v.3.11) and the SciPy library (v.1.11.4), revealed a significant difference in their relative frequencies (U = 7905.0, p < 0.001), confirming that Formicidae was significantly more prevalent in the winter food sources of *P. canus*.

## Discussion

The population of *P. canus* inhabiting the central region of South Korea was observed to feed primarily on Formicidae, even during the winter season. This pattern is consistent with previous studies reporting a strong reliance on ants in this species (Angelstam and Mikusiński 1994, Dauber et al. 2006) and with findings from Hokkaido, Japan, where Formicidae also constituted the main winter food source (Matsuoka and Kojima 1985). Formicidae encompasses ecologically diverse groups, ranging from ground-nesting ants inhabiting crevices to wood-nesting taxa associated with decaying trees and such variation may influence their winter availability to predators, particularly under snow cover. However, because genus-level identification was generally not feasible from the fragmented faecal material, detailed comparisons amongst ant genera were beyond the scope of this study and should be interpreted with caution.

Following Formicidae in relative frequency, Anacardiaceae emerges as a significant food source not only for *P. canus*, but also for various woodpecker species, including the White-backed Woodpecker (*Dendrocopos leucotos*; Matsuoka (1986)), Nuttall’s Woodpecker (*Dryobates nuttallii*; Miller and Bock (1972)), Red-headed Woodpecker (*Melanerpes erythrocephalus*; Williams and Batzli (1979)) and Downy Woodpecker (*Dryobates pubescens*; Kisiel (1972)). Despite producing fruits with less vibrant colours than many other species, Anacardiaceae exhibits colourful foliage in autumn, attracting seed-dispersing birds (Stiles 1982, Facelli 1993). The seeds enclosed within the fruits are coated with nutritious plant-based waxes, providing vital food sources during winter when animal-based food is scarce (Ueda and Fukui 1992). An intriguing aspect is that Anacardiaceae acts as a pioneer plant, forming patches in the early stages of *Pinus* spp. communities, thus attracting birds to facilitate the introduction of deciduous tree seeds (Kamitani et al. 1997). Therefore, the feeding behaviour of *P. canus* feeding on Anacardiaceae fruits may play a crucial role as a contributor to forest succession.

In addition to these dominant food categories, other arthropod groups, such as Hemiptera, Vespidae, Coleoptera and Araneae, were detected only at very low relative frequencies. Their sporadic occurrence likely reflects the limited availability of these taxa during winter or the opportunistic use of such resources when encountered, rather than sustained reliance on these arthropods during the non-breeding season.

*P. canus* maintains its characteristic as an insectivorous bird that persistently feeds on ants even during the winter season. Additionally, our findings confirm the importance of plant-based food sources. The selection of such sources may result from decreased availability of ants or lower energetic demands during the non-breeding season, leading them to prefer easily accessible resources despite potential nutritional deficiencies (Pechacek and Kristin 2004). While shedding light on previously unknown winter food sources of *P. canus*, this study is limited by its somewhat fragmented information. Like *P. canus*, the Black Woodpecker (*Dryocopus martius*) primarily feeds on ants during both breeding and winter seasons. However, ant species differ in seasonal migration strategies and predator responses amongst species, causing *Dryocopus martius* to consume different taxa across seasons (Kojima and Matsuoka 1985). Other woodpecker species, such as *Dryobates pubescens*, Hairy Woodpecker (*Leuconotopicus villosus*) and White-headed Woodpecker (*L. albolarvatus*), also exhibit sex-based variation in ant consumption (Otvos and Stark 1985). These tendencies may also exist in *P. canus*, highlighting the need for further research examining temporal or sex-related variation. Furthermore, because this study was conducted within a single forested region in central South Korea, the results should not be overgeneralised to the species’ broader geographic range, where habitat conditions and winter food availability may differ.
